# Relationship between Serum Ferritin Levels and Dyslipidemia in Korean Adolescents

**DOI:** 10.1371/journal.pone.0153167

**Published:** 2016-04-12

**Authors:** Young-Eun Kim, Do-Hoon Kim, Yong-Kyun Roh, Sang-Yhun Ju, Yeo-Joon Yoon, Ga-Eun Nam, Hyo-Yun Nam, Jun-Seok Choi, Jong-Eun Lee, Jung-Eun Sang, Kyungdo Han, Yong-Gyu Park

**Affiliations:** 1 Department of Family Medicine, Korea University, College of Medicine, Seoul, Republic of Korea; 2 Department of Family Medicine, Hallym University, College of Medicine, Chunchon, Republic of Korea; 3 Department of Family Medicine, Catholic University, College of Medicine, Seoul, Republic of Korea; 4 Department of Biostatistics, Catholic University College of Medicine, Seoul, Republic of Korea; Institute for Health & the Environment, UNITED STATES

## Abstract

**Background:**

Ferritin is associated with various cardiometabolic risk factors such as dyslipidemia, hypertension, obesity, and insulin resistance in adults. We aimed to study the association between serum ferritin levels and dyslipidemia in adolescents, because dyslipidemia is considered an important modifiable cardiovascular risk factor in the young.

**Methods:**

We analyzed 1,879 subjects (1,026 boys and 853 girls) from the 2009–2010 Korean National Health and Nutrition Examination Survey IV. Subjects were categorized into quartiles according to their lipid parameters, which were classified according to age and gender. Those in the highest quartile groups for total cholesterol, low-density lipoprotein cholesterol (LDL-C), and triglyceride concentrations were diagnosed as having dyslipidemia. Those in the lowest quartile for high-density lipoprotein cholesterol (HDL-C) values were diagnosed with abnormal levels.

**Results:**

In boys, total cholesterol, LDL-C, and triglyceride concentrations were significantly correlated with serum ferritin levels. In both boys and girls, serum ferritin levels were negatively associated with HDL-C values, even after adjusting for all covariates. Furthermore, there was no significant correlation between serum ferritin levels and total cholesterol, LDL, and triglyceride concentrations in girls.

**Conclusion:**

Serum ferritin levels were significantly associated with major dyslipidemia parameters, more prominently in boys than in girls, and this association represents a cardiometabolic risk factor.

## Introduction

Ferritin, a ubiquitous intracellular protein essential for the regulation of iron homeostasis, is a clinical biomarker widely used to estimate body iron status. Iron is a transition metal capable of causing oxidative stress and tissue damage by catalyzing the formation of free radicals [1]. Increasing evidence has shown that moderately increased iron stores, resulting in elevated serum ferritin levels, are associated with various metabolic risk factors [2].

Previously, several studies including large cross-sectional studies have shown that increased serum ferritin levels in adults are associated with central obesity [3,4], metabolic syndrome (MS) [5–8], dyslipidemia [9], hypertension [10], insulin resistance [11,12], diabetes mellitus [7,13], and nonalcoholic fatty liver disease [14].

In particular, dyslipidemia, defined as excessive lipids in the blood, may begin in childhood, and is a causal factor of early atherosclerosis and premature cardiovascular diseases (CVD) in young adults [15–18]. Moreover, some studies have demonstrated an association between smoking status, hypertension, obesity, atherosclerosis and dyslipidemia in children and adolescents as well as in adults [19,20].

The levels of lipid parameters and the detection rate of dyslipidemia have increased among children and adolescents in Asian countries including South Korea [21–23]. This trend appears to have been caused by the adoption of a more Westernized diet rich in fat, sugar, and cholesterol following recent economic development [21–23]. Because dyslipidemia is considered an important modifiable risk factor for CVD [16,17], the relationship between dyslipidemia and serum ferritin levels may serve as a predictive factor for cardiometabolic disease in adolescents. While a previous study has shown an association between serum ferritin levels and obesity in Korean male adolescents [24], few studies have examined the relationship between serum ferritin levels and dyslipidemia. Therefore, the aim of this study was to investigate the association between serum ferritin levels and dyslipidemia in South Korean adolescents.

## Methods

### Survey overview and study subjects

This study was based on data obtained from the 2009–2010 Korean National Health and Nutrition Examination Survey (KNHANES), the third year of the KNHANES IV (2007–2009) survey, and the first year of the KNHANES V(2010–2012) survey. The KNHANES conducted by the Korean Ministry of Health and Welfare. This survey was a nationwide representative study that used a stratified, multistage, probability sampling design for the selection of household units and consisted of 3 components: the health interview, nutrition, and health examination surveys.

**Fig 1:**
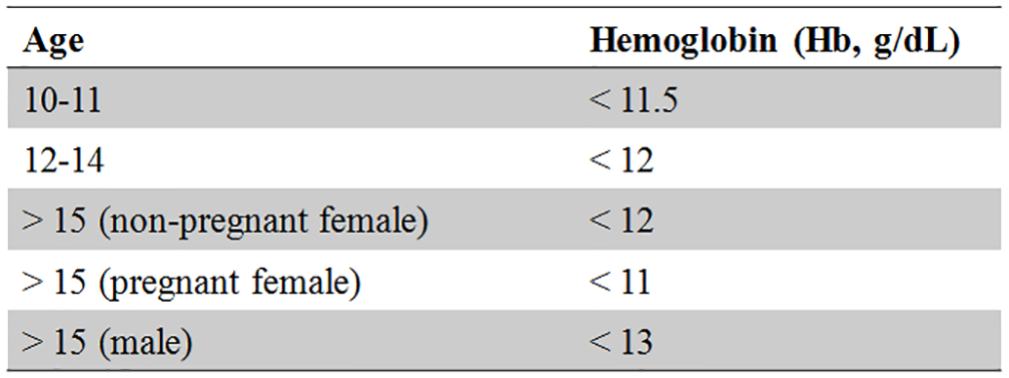
Standard anemia cutoff values associated with age.

**Fig 2:**
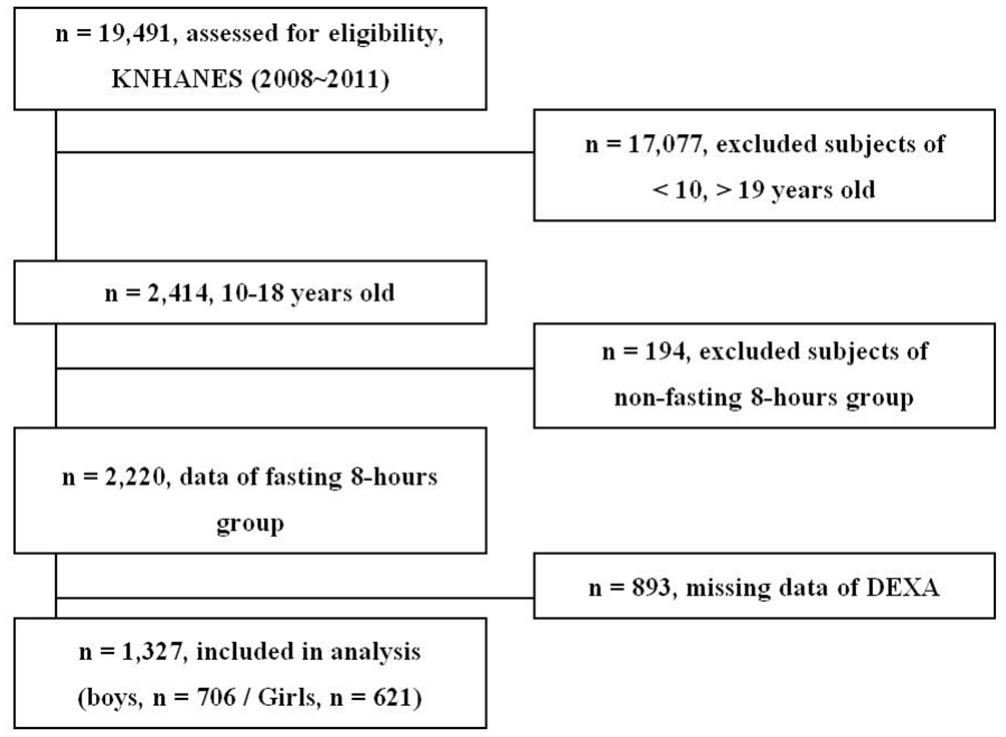
Flow diagram showing the inclusion and exclusion criteria.

A total of 19,491 people participated in the 2009–2010 KNHANES. The present analysis was restricted to 2,414 adolescents aged 10–18 years; 5 individuals who had chronic hepatitis B virus were excluded. The standard anemia cutoff values associated with age were applied to exclude adolescents with anemia or iron deficiency (Fig 1). Consequently, 64 participants were not included in the study. Finally, 466 subjects with missing data were also excluded. The total number of participants eligible for inclusion in the final analysis was 1,879 (1,026 boys and 853 girls; Fig 2).

This study was conducted according to the principles of Helsinki and the study protocol was reviewed and approved by the KCDC (Korea Centers for Disease Control and Prevention)’s institutional review board (IRB approval number: 2009-01CON-03-2C, 2010-02CON-21-C). Written informed consent was obtained from all subjects. The Institutional Review Board of the KCDC approved the study protocol.

### Sociodemographic and lifestyle variables

The sociodemographic and lifestyle variables that were regarded as confounding factors in this study were age, alcohol use, smoking status, physical activity, household income level, and menarche in girls. Health survey of alcohol use, smoking status, physical activity was done by self-reported questionnaire. Questionnaires for alcohol use and smoking status include the evaluation of frequency, quantity, dependence and addiction. Based on drinking of alcohol within one year, subjects were classified as drinkers and non-drinkers. Also, subjects were categorized as non-smokers or smokers according to smoking within a month. Participants recalled their level of physical activities for a week by the days and the duration. Subjects were categorized into 3 groups according to their physical activity levels as follows: sedentary, < 600 metabolic equivalents (METs)/week; minimally active, 600–3000 METs/week; and health-enhancing physical activity, > 3000 METs/week (Sedentary; walking more than 10minutes/ minimally active; Slowly swimming, tennis doubles, volleyball, badminton, table tennis, carrying light weight objects / health-enhancing physical activity; Jogging, hiking, fast bike riding, fast swimming, football, basketball, jump rope, squash, tennis singles, carrying heavy objects) [25]. Monthly household income level was divided into 2 groups: those in the lowest quartile and those above the lowest quartile.

### Anthropometric and biochemical measurements

Height and weight measurements were performed with the participants wearing light clothing and no shoes. The body mass index (BMI) of the participants was calculated as their weight in kilograms divided by the square of their height in meters. Blood pressure was measured with subjects in the sitting position following a 5-minute rest period. Blood pressure was measured on the right arm on 3 occasions with a mercury sphygmomanometer and the final 2 blood pressure readings were averaged. Waist circumference was measured once and it was measured midway between the costal margin and iliac crest at the end of a normal expiration. Blood samples were obtained in the morning following an overnight fast. The serum concentrations of fasting blood glucose (FBG), total cholesterol (TC), high-density lipoprotein cholesterol (HDL-C), low-density lipoprotein cholesterol (LDL-C), and triglyceride (TG) were measured enzymatically using a Hitachi automatic analyzer 7600 (Tokyo, Japan). Serum ferritin, 25-hydroxyvitaminD (25[OH]D) and insulin levels were measured by immunoradiometric assays using a 1470 Wizard gamma-counter (Perkin-Elmer, Turku, Finland). Blood Hb levels were measured using the XE-2100D (Sysmex, Tokyo, Japan). All clinical analyses were performed by the Neodin Medical Institute, a laboratory certified by the Korean Ministry of Health and Welfare.

### Nutritional assessment

Daily nutritional intakes were assessed by the 24-h recall method. The 24-hour-recall foods were recorded for 24 hours prior to the study by once. The 24h recalls were spread throughout the week including week and weekend days. Participants filled out food intake questionnaires for 24 hours prior to the study including food name, weight, volume, materials, manufacturers, whether processed or not. Information of food materials accessed by eye measurement and the volume measuring tools was used to increase the accuracy in the intake investigation. The information was based on the memories of participants but written by using unified measuring tools [26]. Daily intake of total energy, protein, fat and calcium were calculated by using a food database developed for the KNHANES and the food composition table published by the National Rural Living Science Institute under the Rural Development Administration.

### Definition of dyslipidemia and insulin resistance of adolescents

Subjects were categorized into quartiles by their lipid parameters, which were classified according to age and gender. For TC, LDL-C, and TG concentrations, those in the highest quartile were defined as the dyslipidemia group, and for HDL-C values, those in the lowest quartile were assigned to the low HDL-C group. The homeostasis model assessment of insulin resistance (HOMA-IR) was calculated by following equation: $\text{HOMA}-\text{IR }=\;\frac{\text{Fasting insulin }\left(\text{mU}/\text{mL}\right)\times \text{ fasting glucose }\left(\text{mg}/\text{dL}\right)}{405}.$

Participants were categorized into quartile groups according to HOMA-IR and FSI levels with age-specific cutoff values for boys and girls. The values were based on the 2009–2010 KNHANES survey.

### Statistical analysis

General baseline characteristics were presented as the mean ± standard error (SE) or as a percentage ±SE. The differences in baseline characteristics between the boys and girls were assessed by using the Student’s *t*-test for continuous variables or the chi-square test for categorical variables. Multivariable linear regression analysis was performed to assess the correlation between ferritin levels, and dyslipidemia parameters and insulin resistance in 3 multivariable adjusted models. Multivariable logistic regression analysis was used to determine odds ratios (ORs) and 95% confidence intervals (CIs) for dyslipidemia in accordance with serum ferritin levels. Model 1 was adjusted for age. Model 2 was adjusted for height, weight, and age. Model 3 was adjusted for age; height; weight; place; smoking status; drinking; physical activity; serum 25(OH)D levels; total daily energy intake; fat, protein, calcium intakes; and menarche in girls. For each dyslipidemic parameter, the ORs for the highest tertile group of serum ferritin levels were calculated by using the lowest tertile as a reference. Serum ferritin levels were estimated by analysis of covariance (ANCOVA) after adjusting for age, height, and weight, following consideration of the number of dyslipidemic parameters satisfied. The SAS survey procedure (version 9.2; SAS Institute, Cary, N.C., U.S.A.) was used for statistical analyses. P < 0.05 was considered statistically significant.

## Result

### Baseline characteristics of study subjects

The clinical features of the study participants are listed in Table 1 of the 1,879 participants, 853 (45.4%) were girls. There were no significant differences between the mean ages of the girls and boys (P = 0.135). Compared with the girls, the boys had higher mean values for Hb, hematocrit, serum ferritin, height, weight, BMI, systolic and diastolic blood pressure, aspartate aminotransferase, and alanine aminotransferase (P < 0.001 for each value). The levels of total cholesterol, LDL-C, and HDL-C were all higher in girls (P < 0.001). However, there was no gender difference in TG levels. Furthermore, fasting insulin and HOMA-IR were all higher in girls (P < 0.05).

**Table 1:** Principal clinical characteristics of Korean adolescents in the 2009–2010 Korean National Health and Nutrition Examination Survey.

	Boys	Girls	P-value*
	(n = 1026)	(n = 853)	
Place (Rural,yes, %) †	83.6(2.7)	82(3.4)	0.492
Smoking (yes, %)	19.3(1.6)	6.6(1.2)	<0.001
Alcohol intake (yes, %)	25.9(1.7)	17.6(2)	0.002
Physical activity (%)**			<0.001
Sedentary	31.6(1.6)	43.6(2.1)	
Minimally active	41.6(1.9)	44.5(2.1)	
Health-enhancing activity	26.8(1.6)	11.8(1.5)	
Monthly income (lowest quartile, %)	14.5(1.6)	14.3(1.9)	0.912
Menarche (yes)	NA	76.3(2)	.
Age (years)	14.2±0.1	14±0.1	0.135
Height (cm)	165.4±0.5	157.3±0.4	<0.001
Weight (kg)	58.1±0.6	50.3±0.5	<0.001
BMI (kg/m^2)^	21±0.1	20.2±0.2	0.001
SBP (mmHg)	110±0.4	104.2±0.4	<0.001
DBP (mmHg)	68.9±0.4	66.8±0.4	<0.001
FBG (mg/dL)	89.3±0.3	88.3±0.3	0.002
TC (mg/dL)	152.9±1.2	163.1±1	<0.001
LDL-C (mg/dL)	83.9±1.1	90.2±0.9	<0.001
HDL-C (mg/dL)	52.1±0.4	55.5±0.5	<0.001
TG (mg/dL)	73.7(70.6–77)	77.2(74.2–80.3)	0.084
25(OH)D_3_ (ng/mL)	17.1±0.3	16.3±0.3	0.006
FSI (μIU/mL)	12(11.7–12.4)	12.9(12.4–13.4)	0.005
HOMA-IR	2.6(2.6–2.7)	2.8(2.7–2.9)	0.031
Hemoglobin (g/dL)	14.6±0	13.4±0	<0.001
Hematocrit (%)	43.3±0.1	40.1±0.1	<0.001
Serum ferritin (ng/mL)	42.7(40.7,44.9)	26.9(25.4,28.6)	<0.001
ALT (IU/L)	14.7(14.1–15.3)	10.9(10.6–11.2)	<0.001
AST (IU/L)	19.4(19–19.8)	17(16.7–17.3)	<0.001
Daily energy intake (kcal)	2290.9±39.3	1843.6±35.7	<0.001
Daily protein intake (%)	14.3±0.2	13.7±0.2	0.013
Daily fat intake (%)	22.6±0.3	22.6±0.4	0.923
Daily intake of calcium (mg)	539.4±15.7	450.6±13.8	<0.001

Abbreviations: BMI, body mass index; SBP, systolic blood pressure; DBP, diastolic blood pressure; FBG, fasting blood glucose; TC, total cholesterol; LDL-C, low-density lipoprotein-cholesterol; HDL-C, high-density lipoprotein cholesterol; TG, triglyceride; 25(OH)D_3_, 25-hydroxyvitamin D_3;_ FSI, fasting serum insulin; HOMA-IR, homeostasis model assessment of insulin resistance; ALT, alanine transaminase; AST, aspartate transaminase; NA, not applicable.

Data are presented as mean ± standard error (SE) or percentage ±SE.

TG, AST, ALT, FSI levels, and HOMA-IR were tested after logarithmic transformation.

* P-values were obtained by using the chi-square test or Student’s *t*-test.

** Study subjects were categorized according to their physical activity levels as follows: sedentary, < 600 metabolic equivalents (METs)/week; minimally active, 600–3000METs/week; and health-enhancing physical activity, > 3000METs/week.

^†^ Place means the area of residence and was categorized into rural and urban area. Urban area included metropolitan cities and province such as ward and county were classified into rural area.

### Relationship of serum ferritin level with lipid parameters and HOMA–IR

Table 2 shows the relationship of serum ferritin levels with lipid parameters and HOMA-IR. In boys, total cholesterol, LDL-C, and TG concentrations were positively correlated with serum ferritin levels. HDL-C values were negatively associated with serum ferritin levels in both boys and girls. In girls, there was no positive correlation between serum ferritin levels and TC, LDL, or TG concentrations. There was no significant statistical association between HOMA-IR and any of the lipid parameters in the multivariable-adjusted models.

**Table 2:** Multivariable linear regression analysis to determine the association of serum ferritin levels with lipid parameters and the homeostasis model assessment of insulin resistance by gender.

Response variable	Model	Boys			Girls		
		B	SE	P-value*	B	SE	P-value*
TC	Model 1	9.783	2.002	<0.001	-0.327	1.648	0.843
	Model 2	8.7	1.964	<0.001	-0.45	1.588	0.777
	Model 3	8.36	2.014	<0.001	-0.676	1.67	0.686
HDL-C	Model 1	-2.55	0.724	0.001	-2.115	0.704	0.003
	Model 2	-2.129	0.695	0.002	-2.193	0.64	<0.001
	Model 3	-2.137	0.695	0.002	-2.116	0.657	0.002
LDL-C	Model 1	10.126	1.72	<0.001	1.235	1.415	0.384
	Model 2	9.203	1.688	<0.001	1.173	1.292	0.365
	Model 3	9.043	1.726	<0.001	0.815	1.365	0.551
TG**	Model 1	0.124	0.037	0.001	0.007	0.033	0.824
	Model 2	0.095	0.037	0.012	0.008	0.031	0.804
	Model 3	0.085	0.037	0.023	0.011	0.032	0.737
HOMA-IR**	Model 1	0.02	0.03	0.508	0.008	0.035	0.819
	Model 2	-0.022	0.026	0.385	0.013	0.03	0.664
	Model 3	-0.021	0.026	0.419	0.018	0.03	0.562

Abbreviations: B, unstandardized regression coefficient; SE, standard error; TC,total cholesterol; LDL-C, low-density lipoprotein-cholesterol; HDL-C, high-density lipoprotein cholesterol; TG, triglyceride; HOMA-IR, homeostasis model assessment of insulin resistance.

* P-values were obtained by multivariable linear regression analyses after adjusting for age (Model 1); age, height, and weight (Model 2); and age, height, weight, place, smoking status, drinking, physical activity, serum 25(OH)D level, total daily energy intake, fat intake, protein intake, calcium intake, and menarche in girls (Model 3).

** TG levels and HOMA-IR were tested after logarithmic transformation.

### Adjusted ORs and 95%CIs for dyslipidemia according to serum ferritin levels

Table 3 shows the adjusted ORs and 95% CIs for dyslipidemic parameters according to each increase in log-transformed serum ferritin levels. In boys, the ORs for high TC, high LDL-C, and low HDL-C concentrations were significantly increased in the highest tertile group of serum ferritin levels. The ORs for high TG values in the highest tertile group of serum ferritin levels were also significantly increased in Models 1 and 2, but not in Model 3. However, this trend was not observed in girls. Only the ORs for low HDL-C values were significantly increased in all multivariable-adjusted models in both genders. The ORs for high HOMA-IR were only significant in Model 1 in boys.

**Table 3:** Adjusted odds ratios for dyslipidemic parameters according to each increase in log-transformed serum ferritin levels.

Model	Response variables	Boys		Girls	
		OR (95% CI)*	P-value	OR (95% CI)*	P-value
Model 1	High TC	1.733(1.321–2.274)	< 0.001	0.948(0.687–1.308)	0.745
	Low HDL-C	1.917(1.387–2.648)	< 0.001	1.793(1.336–2.406)	<0.001
	High LDL	2.053(1.564–2.694)	< 0.001	1.246(0.917–1.693)	0.16
	High TG	1.558(1.137–2.135)	0.006	0.987(0.722–1.35)	0.936
	High HOMA-IR	1.420(1.035–1.948)	0.03	0.939(0.682–1.294)	0.702
Model 2	High TC	1.529(1.159–2.017)	0.003	0.937(0.687–1.276)	0.679
	Low HDL-C	1.786(1.268–2.515)	0.001	1.828(1.341–2.492)	<0.001
	High LDL	1.824(1.374–2.422)	< 0.001	1.231(0.919–1.649)	0.164
	High TG	1.409(1.022–1.941)	0.036	0.961(0.714–1.295)	0.794
	High HOMA-IR	1.124(0.805–1.567)	0.493	0.888(0.635–1.243)	0.489
Model 3	High TC	1.485(1.065–2.07)	0.02	0.901(0.655–1.238)	0.519
	Low HDL-C	1.636(1.124–2.379)	0.01	1.866(1.308–2.662)	<0.001
	High LDL	1.882(1.337–2.648)	< 0.001	1.144(0.840–1.558)	0.393
	High TG	1.299(0.908–1.859)	0.153	1.161(0.853–1.580)	0.342
	High HOMA-IR	0.926(0.606–1.414)	0.722	1.002(0.694–1.446)	0.991

Abbreviations: OR, odds ratio; CI, confidential intervals; TC, total cholesterol; LDL-C, low-density lipoprotein-cholesterol; HDL-C, high-density lipoprotein cholesterol; TG, triglyceride; HOMA-IR, homeostasis model assessment of insulin resistance.

* Odds ratios and 95% confidence intervals were obtained by multivariable logistic regression analysis after adjusting for age (Model 1); age, height, and weight (Model 2); and age, height, weight, place, smoking status, drinking, physical activity, serum 25(OH)D level, total daily energy intake, fat intake, protein intake, calcium intake, and menarche in girls (Model 3).

### Estimated serum ferritin levels according to the number of dyslipidemic parameters

**Fig 3:**
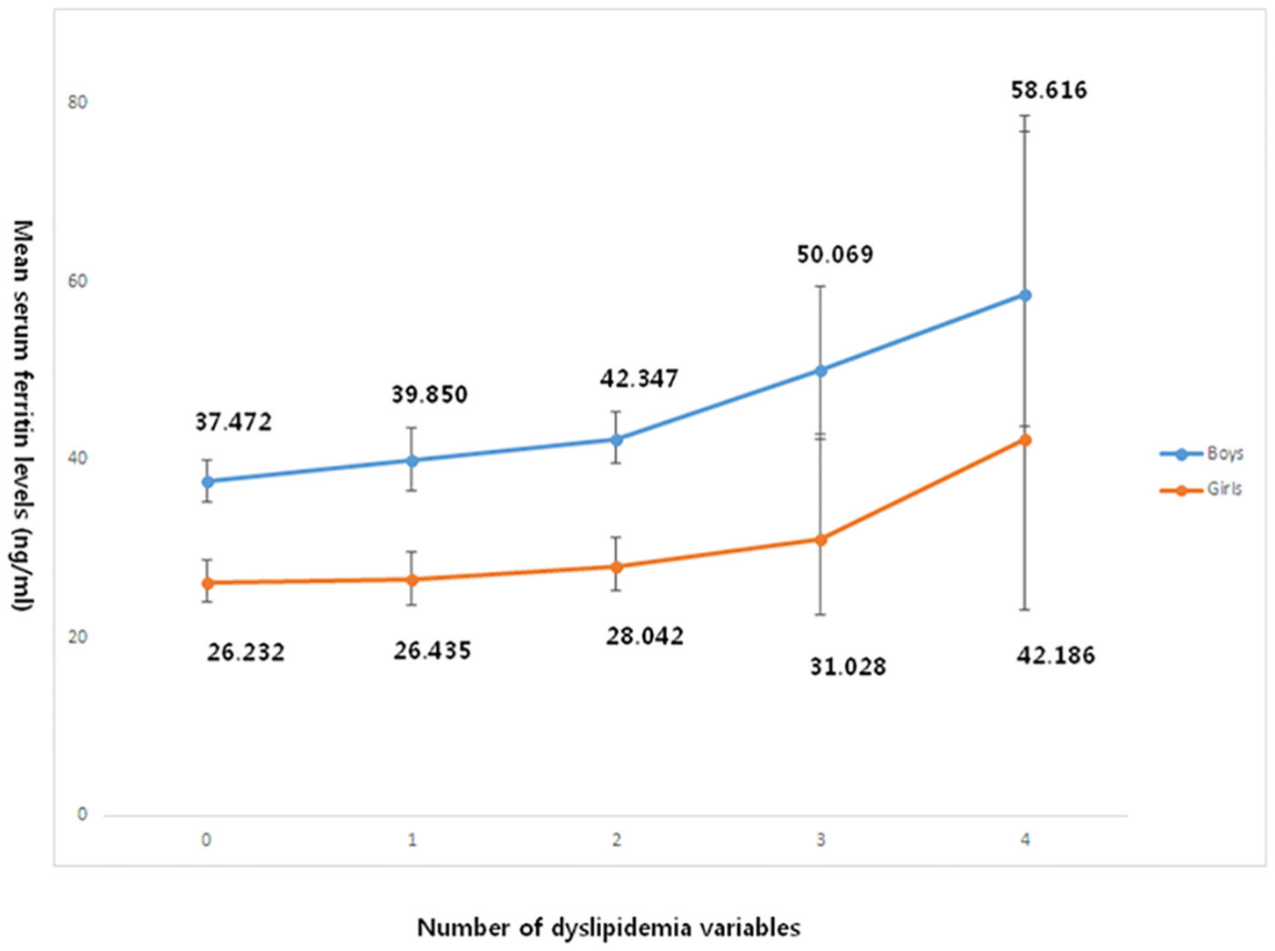
Analysis of covariance of serum ferritin levels according to the number of satisfied dyslipidemia variables in age-, height-, weight-, place-, smoking status-, drinking-, physical activity-, serum 25(OH)D levels-, total daily energy intake-, fat-, protein-, calcium intakes; and menarche in girls-adjusted model (boys, P < 0.001; girls, P = 0.06).

Fig 3 shows the trend in serum ferritin levels according to the number of satisfied dyslipidemic parameters, which were estimated by ANCOVA after adjustments for age, height, weight, place, smoking status, drinking, physical activity, serum 25(OH)D levels, total daily energy intake, fat, protein, calcium intakes and menarche in girls. The mean serum ferritin levels significantly increased as the number of satisfied dyslipidemic parameters accumulated in both genders (boys, P < 0.001; girls, P < 0.06).

## Discussion

The major finding of this study was that serum ferritin levels were associated with prevalent dyslipidemia in a large cross-sectional study of Korean adolescents from the 2009–2010 KNHANES. There was a strong positive association between TC, LDL-C, and TG, and serum ferritin levels in boys, while there was a significant negative correlation between HDL-C and serum ferritin levels in both genders.

As a protein of iron-storage, the serum ferritin is a tool for the evaluation of common disease states, such as iron deficiency anemia. And also it is able to evaluating hereditary and acquired iron overload conditions, such as hereditary hemochromatosis, thalassemias, hemoglobinopathy and chronic transfusion therapy [27]. So, diseases that might affect the iron storage are possible determinant of serum ferritin levels. Furthermore serum ferritin is also recognized as an acute phase marker of inflammation such as chronic kidney disease [28], rheumatoid arthritis and other autoimmune disorders [29]. And many studies had shown the serum ferritin as a determinant of myocardiac ischemia [30], metabolic syndrome [31], Non-alcoholic fatty liver disease and hyperinsulinemia [32].

In addition, iron is a transition metal capable of causing oxidative stress-induced tissue damage by catalyzing the conversion of hydrogen peroxide to free radicals that attack cellular membranes, proteins, and DNA [1,8,12,33], leading to derangements in glucose homeostasis such as insulin resistance and pancreatic β-cell dysfunction through iron toxicity [34,35]. Chronic oxidative stress is associated with disruption of mitochondrial β-oxidation of long chain fatty acids and β-cell dysfunction in the pancreas [36,37]. Abnormal mitochondrial β-oxidation leads to hypertriglyceridemia, and excessive triglyceride accumulation in muscle and liver tissue, while pancreatic β-cell dysfunction results in hyperglycemia due to insufficient insulin production [36,37].

Previously, several studies have suggested apositive relationship between serum ferritin levels and metabolic risk factors in adults. Kang et al. reported that increased serum ferritin levels were associated with high TG and FBG concentrations in men and women [5]. Yoo et al. confirmed that low HDL-C values and MS were associated with high serum ferritin levels in Korean non-obese young women [8]. Similarly, Lee et al. suggested that high serum ferritin levels were linked to MS in women and to diabetes mellitus in men and premenopausal women [7]. Moreover, in 2004, Jehn et al. conducted a cross-sectional study of 6,044 U.S. adults. In this study, they observed a positive correlation between serum ferritin levels and the prevalence of MS components such as TG and plasma glucose in men and women [6]. In 2013, in China, Li et al. showed an association between serum ferritin levels and MS in 8,441 Chinese adults, ≥ 18 years. MS was most prevalent in men and women in the highest quartile group for serum ferritin levels, and included those with high TG and low HDL-C concentrations [38]. Chang et al. also suggested that high serum ferritin levels were significantly associated with FBG and TG values [39]. Our research extended these previous results by studying adolescents.

Although some large cross-sectional studies have suggested an association between ferritin levels, and dyslipidemia and some metabolic diseases, few studies have focused on adolescents. Recently, Lee et al. reported that children in a group with high HOMA-IR had greater total iron-binding capacity and serum transferrin levels, and were more likely to develop MS, butthey could not demonstrate an association between serum ferritin levels and dyslipidemia parameters [40]. A study byJeon et al. also examined dyslipidemia parameters in Korean adolescents, and observed a link between serum ferritin levels and obesity, but not between serum ferritin levels and MS components [24]. In contrast to previous studies, we observed an association between serum ferritin levels and dyslipidemia, especially in boys.

Interestingly, the present study identified a gender difference in the association of serum ferritin levels with lipid parameters. In boys, dyslipidemia parameters such as TC, LDL-C, HDL-C, and TG were all significantly associated with serum ferritin levels. Conversely, in girls, serum ferritin levels were only significantly associated with low HDL-C concentrations. Gender differences were seenin other studies on adults previously. However, many studies have suggested that serum ferritin levels appear to be correlated with metabolic risk factors or disease in women rather than men [7,8]. In agreement with our study, Han et al. showed that elevated serum ferritin levels were positively associated with MS, obesity, and diabetes in men, but not in women [41]. Although the underlying cause of gender differences in adolescents is unclear, intrinsic sexual dimorphisms at the molecular and cellular levels, and the effects of different sex steroid hormones may play a part [42]. In addition, the transient physiological increase in insulin resistance during puberty could lead to increased activity of the insulin-like growth factor-I/growth hormone axis [43] or sex hormones [44]. In adults, men have more visceral fat accumulation and lower plasma adiponectin, which contribute to gender differences in insulin sensitivity and vulnerability to cardiovascular disease [45]. Women are intrinsically more insulin resistant than men, and this may be due to specific sex-linked gene expression which affects metabolic control elements (e.g., signaling pathway and substrate shuttling elements, and receptors). Sex hormones, and environmental and life-style factors augment women’s susceptibility, in ways that may be genetically predetermined [42].

The present study has several limitations. First, because we used a cross-sectional study design, it was difficult to examine the causal relationship between serum ferritin levels and dyslipidemia. Second, we could not exclude subjects with active infection or inflammation, or those with elevated inflammatory markers such as white blood cell counts or C-reactive protein. These could be potential confounding factors and affect our results. Third, serum iron, transferrin, and total iron-binding capacity were not measured in conjunction with serum ferritin levels in the 2009–2010 KNHANES. Fourth, regarding alcohol consumption, no dose related effect can be taken into account as only a dichotomous variable was coded, classifying subjects as drinkers or non-drinkers. The same applies to smoking status and it could be the limitation of this study. Despite these limitations, it is worth noting that this study was conducted using nationwide representative data from a survey based on a stratified, multistage, probability sampling design. Furthermore, few studies have examined the relationship between serum ferritin levels and metabolic risk factors in adolescents. To our knowledge, this study is the first to report an association between serum ferritin levels and various dyslipidemia parameters in adolescents using recent nationally representative data.

In conclusion, serum ferritin levels were significantly associated with major dyslipidemia parameters, more prominently in boys than in girls, and this association represents a cardiometabolic risk factor.

## Abbreviations

ALT: alanine aminotransferase
ANCOVA: analysis of covariance
AST: aspartate aminotransferase
BMI: body mass index
CI: confidence interval
CVD: cardiovascular diseases
FBG: fasting blood glucose
Hb: hemoglobin
HDL-C: high-density lipoprotein cholesterol
HOMA-IR: homeostasis model assessment of insulin resistance
KCDC: Korean Centers for Disease Control
KNHANES: Korea National Health Examination and Nutrition Survey
LDL-C: low-density lipoprotein cholesterol
METs: metabolic equivalents
MS: metabolic syndrome
OR: odds ratio
SE: standard error
TC: total cholesterol
TG: triglyceride
25(OH)D: 25-hydroxyvitamin D
